# Exercise modulation of gut microbiota in Alzheimer’s disease: pathophysiological mechanisms and therapeutic perspectives

**DOI:** 10.3389/fnagi.2025.1677896

**Published:** 2025-11-07

**Authors:** Si-yi Leng, Qi-hang Yang, Yang Yuan, Bingao Chen, Hongbao Chen, Weikang Ban, Jiahao Zhang

**Affiliations:** 1 Center for Active Aging and Health Research, School of Physical Education and Sports Science, Qufu Normal University, Qufu, China; 2 Cancer Institute, The Affiliated Hospital of Qingdao University, Qingdao, China

**Keywords:** exercise, gut microbiota, Alzheimer’s disease, gut-brain axis, neuroinflammation, blood-brain barrier, cognitive function

## Abstract

As global life expectancy increases, Alzheimer’s disease (AD) has become a major public health concern. The gut microbiota plays a pivotal role in regulating the central nervous system, influencing both behavior and cognitive functions in AD through direct and indirect mechanisms. Physical exercise has been shown to positively modulate the diversity and composition of the gut microbiota, emerging as a significant factor in slowing AD progression. A growing body of research highlights the dynamic interactions between exercise, gut microbiota, and AD, revealing that exercise can alter the synthesis and metabolism of key neuroactive substances, such as glutamate and aspartate, thereby enhancing cognitive function. Moreover, exercise influences peripheral and central immune responses via microbiota modulation, reducing neuroinflammation, amyloid-β (Aβ) deposition, and tau phosphorylation. Exercise also regulates gut microbiota-derived metabolites, including short-chain fatty acids (SCFAs), which are crucial for alleviating neuroinflammation and maintaining the integrity of the blood-brain barrier (BBB). This review synthesizes recent advances in the molecular mechanisms underpinning the exercise-microbiota-AD axis, offering new therapeutic perspectives for AD.

## 1 Introduction

Alzheimer’s disease (AD) is a multifactorial neurodegenerative disorder characterized by cognitive decline, including memory impairment and behavioral abnormalities. The onset of AD is driven by complex interactions among genetic, molecular, and environmental factors (Lane et al., 2017). According to the World Health Organization, AD is rapidly becoming a global public health challenge, with projections indicating that by 2050, more than 131 million people will be affected worldwide (Scheltens et al., 2021). Key pathological features of AD include amyloid-β (Aβ) accumulation, tau hyperphosphorylation, neuronal and synaptic dysfunction, and neuroinflammation, all of which contribute to disease progression (Rostagno, 2022). Early research primarily focused on the gut microbiota as a potential biomarker for diagnosing AD. However, more recent studies have elucidated a bidirectional regulatory relationship between the gut microbiota and the brain via the gut-brain axis (Rutsch et al., 2020). Dysbiosis of the gut microbiota has been implicated in neuronal damage, triggering immune system dysfunction and neuroinflammation, which compromise the blood-brain barrier and heighten the risk of AD development (Wu et al., 2021).

Physical exercise has long been recognized as a critical strategy for promoting both physical and mental health. Emerging evidence now highlights the complex and dynamic interplay between exercise and the gut microbiota (Dohnalová et al., 2022). The gut microbiota comprises a diverse community of microorganisms inhabiting the gastrointestinal tract, including bacteria, viruses, and fungi. At the phylum level, the gut microbiota is dominated by Bacteroidetes, Firmicutes, and Proteobacteria, while at the genus level, *Bacteroides*, *Prevotella*, *Roseburia*, and *Lachnospira* are prominent (Adak and Khan, 2018). The composition of the gut microbiota is influenced by various factors, including diet, physiological stress, environmental exposures, and intestinal infections (Bell, 2015). Importantly, exercise has emerged as a key modulator of gut microbiota composition. A growing body of evidence suggests that physical activity plays a vital role in reducing the risk and onset of neurodegenerative diseases such as Alzheimer’s, Parkinson’s, and amyotrophic lateral sclerosis, all of which are associated with gut dysbiosis.

Due to the multifactorial and heterogeneous nature of AD, a definitive cure remains elusive, and current drug treatments only offer symptomatic relief, often with potential side effects. Research has identified physical inactivity as a significant risk factor for AD, with a lack of exercise during adolescence increasing the likelihood of developing dementia later in life (Middleton et al., 2010). In contrast, physical activity has been shown to reduce the risk of both dementia and AD (Lin et al., 2018). Moreover, engaging in targeted physical activity interventions and adopting healthy lifestyle habits during the stages of mild cognitive impairment and pre-dementia can delay the onset of dementia in approximately one-third of global cases (Rosa et al., 2020).

Numerous studies have elucidated the complex relationship between the gut microbiota and AD, indicating that alterations in the composition and functionality of the gut microbiota may play a pivotal role in the onset and progression of AD (Mou et al., 2022). New investigations highlight the dynamic interactions between physical exercise, the gut microbiota, and neurodegenerative diseases (Monda et al., 2017; Rojas-Valverde et al., 2023). However, the exact mechanisms underlying these interactions—both individually and collectively—remain poorly understood. This article aims to provide an integrated perspective on how physical exercise influences the physiological pathways linking the gut microbiota and the brain. Initially, the article reviews the molecular mechanisms by which the gut microbiota contributes to AD pathophysiology through neuroendocrine signaling, modulation of peripheral and central immune systems, and the production of its metabolites. It then explores how different types of exercise can reshape the gut microbiota, fortify gut barrier integrity, and attenuate inflammatory responses. Moreover, moderate exercise has been shown to alleviate AD by mitigating neuroinflammation, reducing Aβ deposition, and regulating tau hyperphosphorylation, while excessive exercise may exert detrimental effects on the central nervous system. Finally, the article discusses the molecular mechanisms through which exercise-induced modulation of the gut microbiota may serve as a preventive and therapeutic strategy for AD, providing essential insights for future research on exercise interventions aimed at targeting the gut microbiota in the context of AD prevention and treatment.

## 2 Materials and methods

### 2.1 Literature search strategy

We conducted a narrative review of the literature to synthesize current knowledge on the effects of physical exercise on gut microbiota and downstream pathways in the context of AD. Searches were performed in PubMed, Web of Science, and Embase databases from their inception until May 2025, using combinations of the following keywords and MeSH terms: exercise, physical activity, training, gut microbiota, intestinal flora, Alzheimer’s disease, cognitive impairment, amyloid, tau, neuroinflammation, blood–brain barrier. Reference lists of included articles were also manually screened to identify additional relevant publications.

### 2.2 Eligibility criteria

Inclusion criteria: (i) human studies, animal AD models, or *in vitro* experiments; (ii) interventions involving structured physical exercise; (iii) outcomes related to gut microbiota composition, microbial metabolites, or AD-relevant endpoints. Exclusion criteria: reviews, editorials, conference abstracts, non-exercise interventions, studies unrelated to gut microbiota–AD pathways, or non-English publications.

### 2.3 Study selection and prioritization

Titles, abstracts, and full texts were independently reviewed by two authors, with discrepancies resolved through discussion. To ensure robustness of evidence, human studies (including randomized controlled trials and longitudinal cohort studies) were prioritized over cross-sectional, animal, and *in vitro* studies during the synthesis of conclusions. Non-Alzheimer’ s disease (AD) models (e.g., Parkinson’ s disease, multiple sclerosis) were included only if they provided direct mechanistic insights relevant to AD and were explicitly identified as such.

### 2.4 Quality appraisal

Although the primary purpose of this review was descriptive synthesis, we applied established appraisal tools when discussing study reliability: SYRCLE risk of bias tool for animal studies, and Newcastle–Ottawa Scale or ROBINS-I for observational human studies. These tools were not used for formal scoring but to contextualize the strength and limitations of the evidence base.

### 2.5 Evidence grading

In the final synthesis, conclusions were stratified according to an evidence hierarchy:

Human randomized controlled trials (highest level)

Human longitudinal cohort studies

Human cross-sectional studies

Animal experiments

*In vitro* models (lowest level)

This stratification is explicitly reflected in the Discussion, where stronger causal inference is attributed to human randomized controlled trials (RCTs), while preclinical studies are used to generate mechanistic hypotheses.

## 3 Gut microbiota and Alzheimer’s disease

### 3.1 Experimental evidence of gut microbiota involvement in AD pathology

Accumulating evidence indicates that alterations in the gut microbiota are involved in the onset and progression of AD.

Clinical investigations have demonstrated that the gut microbiota may serve as potential biomarkers for AD diagnosis and disease monitoring. In particular, an increased abundance of pro-inflammatory genera such as *Escherichia*—*Shigella* and a reduced abundance of anti-inflammatory taxa such as *Eubacterium* have been associated with greater severity of cognitive impairment in AD patients (Cattaneo et al., 2017). Furthermore, transplantation of fecal microbiota from AD patients into germ-free rodents induces cognitive deficits, impaired hippocampal neurogenesis, and memory dysfunction, suggesting a causal contribution of human-derived microbiota to disease-relevant phenotypes (Wang et al., 2022; Grabrucker et al., 2023).

Findings from transgenic and germ-free animal models provide mechanistic insights into the role of gut microbiota in AD pathology. For example, APPswe/PS1dE9 mice exhibit a higher relative abundance of Helicobacteraceae and Desulfovibrionaceae, as well as increased levels of *Odoribacter* and *Helicobacter*, compared to the control group (Shen et al., 2019). Similarly, colonization of healthy C57BL/6 mice with microbiota derived from 5xFAD mice induces colonic inflammation, abnormal microglial activation in the hippocampus, reduced hippocampal neurogenesis, and decreased brain-derived neurotrophic factor (BDNF) expression, ultimately resulting in memory impairments (Kim et al., 2021). Excessive endoplasmic reticulum (ER) stress has also been implicated in this process, as transplantation of microbiota from AD patients or APP/PS1 mice into recipient mice leads to dysbiosis and heightened ER stress in the cerebral cortex, mediated by trimethylamine-N-oxide (TMAO) (Wang et al., 2022).

Conversely, beneficial effects are observed when microbiota from healthy donors are introduced into AD models. Colonization with healthy microbiota reduces tau phosphorylation, enhances synaptic plasticity through the upregulation of postsynaptic density protein-95 and synaptophysin, and decreases Aβ deposition, thereby improving cognitive performance (Sun et al., 2019). Likewise, transplantation from healthy mice modulates intestinal macrophage activity and normalizes circulating cytokine expression, which in turn reduces Aβ accumulation and tau hyperphosphorylation (Kim et al., 2019). Together, these findings underscore the dual role of gut microbiota in either exacerbating or mitigating AD-related neuropathology, depending on microbial composition.

### 3.2 Mechanisms of gut microbiota in Alzheimer’s disease

The gut and brain are functionally connected through various pathways, including neurons of the sympathetic and parasympathetic nervous systems, circulating hormones, neurotransmitters, and immune mediators. This connection forms the mechanistic basis for the gut microbiota’s regulation of central physiological and pathological processes. Consequently, the gut microbiota can modulate the central nervous system, either directly or indirectly, thereby influencing the behavior and cognitive function in AD. Specifically, the gut microbiota participates in the onset and progression of AD through its effects on neuroendocrine regulation (such as learning and memory), immune responses (including neuroinflammation and mood changes), and microbial metabolites [such as TMAO, short-chain fatty acids (SCFAs), and bile acids (BAs)].

#### 3.2.1 Neuroendocrine regulation

Imbalances in central neurotransmitters can contribute to the development of various neurological and psychological disorders, including Alzheimer’s disease, autism spectrum disorders, and depression (Braga et al., 2024). In germ-free (GF) mice, compared with conventionally raised controls, significant alterations have been observed in neurotransmitter levels such as γ-aminobutyric acid (GABA), serotonin, and acetylcholine, as well as their metabolic precursors, including tryptophan and choline, in both fecal and serum samples (evidence primarily from animal and *in vitro* studies) (Wikoff et al., 2009; Clarke et al., 2012; Sjögren et al., 2012; Yano et al., 2015; Matsumoto et al., 2017; Lahiri et al., 2019). Recent studies suggest that the gut microbiota may influence AD by modulating the synthesis and metabolism of key substances, including GABA, glutamate, acetylcholine, and the serotonin precursor tryptophan (evidence primarily from animal and *in vitro* studies).

Gut microbiota-derived GABA is unable to cross the blood-brain barrier and may exert localized effects on the enteric nervous system or via the vagus nerve, thereby influencing the central nervous system (Loh et al., 2024). GABA serves as the primary inhibitory neurotransmitter in the mammalian brain, and its levels are notably lower in individuals with AD compared to healthy controls (Carello-Collar et al., 2023). As a key modulator of central nervous system function, GABA regulates various physiological states within the body. *Escherichia coli* and *Pseudomonas* putida have been shown *in vitro* to reduce gut-derived GABA levels, while *Bacteroides*, *Lactobacillus*, and *Bifidobacterium* can synthesize GABA (*in vitro* mechanistic insight) (Strandwitz et al., 2018; Carello-Collar et al., 2023). Preclinical findings further support a regulatory role of gut microbiota in animal clinical studies further support a regulatory role of gut microbiota in neuroendocrine function. In APP/PS1 AD mice, colonization with *Lactobacillus rhamnosus* has been shown to regulate GABA receptor expression in the amygdala and hippocampus, reduce stress-induced corticosterone levels, and mitigate anxiety- and depression-like behaviors; these effects were abolished after vagus nerve ablation (animal model, causal evidence) (Bravo et al., 2011; Janik et al., 2016). Furthermore, the gut microbiota metabolite acetate supports the glutamate-glutamine shuttle, which in turn promotes the production of lactate and GABA, thereby inducing anorexic signals within the arcuate nucleus of the hypothalamus and contributing to reduced appetite (Frost et al., 2014).

The gut microbiota plays a key role in cognition by regulating glutamate metabolism. Glutamate, a major excitatory neurotransmitter in the brain and an agonist of N-methyl-D-aspartate receptors, is essential for normal cognitive function. However, disruptions in glutamate neurotransmission can lead to neurotoxic damage to neurons, impairing memory and learning (Rothman and Olney, 1986; Choi, 1988). Specific bacterial species such as *Bacteroides* and *Campylobacter jejuni* influence glutamate metabolism by reducing the production of 2-ketoglutarate. Additionally, species containing glutamate decarboxylase, including *Glutamicibacter*, *Lactobacillus fermentum*, and *Bacillus subtilis*, can convert L-glutamate to D-glutamate, thus affecting cognitive function in AD patients (Chang et al., 2020). Other research indicates that an increased abundance of Firmicutes, Bacteroidetes, and *Burkholderia* in the gut of obese individuals is associated with a decrease in glutamate levels and altered metabolism, ultimately contributing to cognitive decline (Palomo-Buitrago et al., 2019). Furthermore, Animal experiments further revealed the interaction between gut microbiota and D-glutamate metabolism. Germ-free mice exhibit elevated levels of D-aspartate, D-serine, and L-serine in certain brain regions compared to specific-pathogen-free mice (Kawase et al., 2017). Enhancing glutamatergic neurotransmission has been shown to inhibit the expression of vesicular glutamate transporter 1 in presynaptic vesicles of AD mice, modulate the glutamatergic/GABAergic balance in the hippocampal dentate gyrus, significantly reduce Aβ and tau protein deposition, suppress glutamate release induced by external stimuli, and improve synaptic plasticity, ultimately restoring cognitive function (Shen et al., 2025).

Acetylcholine is a crucial mediator in both the central and peripheral nervous systems, playing a key role in excitatory signaling between neurons. The cholinergic system plays a critical role in promoting neuronal plasticity (Hampel et al., 2018). Due to its inability to cross the blood-brain barrier, acetylcholine is synthesized within central nervous system neurons from choline and acetyl-CoA, a process catalyzed by choline acetyltransferase (Picciotto et al., 2012). In AD rat models, oral administration of *Lactobacillus* plantarum MTCC 1325 has been shown to increase acetylcholine levels in the hippocampus and cerebral cortex, improving memory and behavioral performance (animal model, causal evidence) (Nimgampalle and Kuna, 2017). Both acetylcholine deficiency and excessive stimulation can induce neuronal apoptosis in AD mice, contributing to short-term memory impairment and exacerbating cognitive deficits (Sun et al., 2022). Furthermore, Supplementation with *Lactobacillus fermentum* LAB9 and *Lactobacillus* casei LABPC has been shown to enhance cholinergic neurotransmission and attenuate neuroinflammation in rodent AD models (animal model, causal evidence) (Musa et al., 2017). Additionally, In healthy human volunteers, Panax ginseng extract supplementation has been reported to improve short-term memory and attention by modulating gut microbiota and upregulating acetylcholine (human interventional study, preliminary evidence) (Bell et al., 2021).

The gut microbiota plays a crucial role in modulating tryptophan metabolism, which in turn regulates central serotonin levels and influences cognitive function. Serotonin in the human body can be categorized into peripheral (gut-derived) serotonin and central serotonin. Since serotonin itself cannot cross the blood–brain barrier, alterations in peripheral serotonin have limited direct impact on central neurotransmission. In contrast, tryptophan, the metabolic precursor of serotonin, is able to cross the blood–brain barrier, and the gut microbiota has been shown to regulate tryptophan availability (Reigstad et al., 2014). In human studies, In AD rat models, supplementation with *Lactobacillus helveticus* NS8 has been reported to elevate hippocampal serotonin levels and upregulate BDNF expression, improving cognitive performance (animal model, causal evidence) (Hang et al., 2022). In animal studies, in germ-free mice, hippocampal serotonin levels are elevated relative to specific-pathogen-free controls, suggesting microbial colonization affects tryptophan metabolism and serotonin homeostasis (animal model, correlational evidence) (Clarke et al., 2012). These findings suggest that microbial colonization affects tryptophan metabolism and thereby modulates central serotonin homeostasis. Additional evidence indicates that butyrate, a short-chain fatty acid, exerts neuroprotective effects in stressed mice by enhancing brain serotonin levels and restoring blood–brain barrier integrity (Sun et al., 2016). Conversely, lipopolysaccharide (LPS), a key component of Gram-negative bacterial membranes, reduces serotonin concentrations in the prefrontal cortex (Zhu et al., 2015). Collectively, these results indicate that gut microbiota–mediated regulation of tryptophan and serotonin metabolism can either impair or protect cognitive function, depending on microbial composition and host context.

#### 3.2.2 Mediating peripheral and central immunity

Gut microbiota dysbiosis can lead to immune system abnormalities, initiating persistent peripheral inflammation, which in turn accelerates blood-brain barrier (BBB) damage and brain inflammation, thereby exacerbating the pathogenesis of AD. The gut microbiota interacts with the host immune system to facilitate the establishment and maintenance of the intestinal barrier. Under conditions of mild dysbiosis, the host immune system typically adjusts to prevent pathogenic shifts in the microbiota (Chung et al., 2012). When dysbiosis exceeds the immune system’s regulatory capacity, it triggers an imbalance in the intestinal immune response, leading to local and systemic inflammation (Cani et al., 2008; Fransen et al., 2017; Tetz et al., 2020).

Dysbiosis can promote the overgrowth of Gram-negative bacteria, leading to the excessive production of LPS. These LPS molecules induce intestinal inflammation and disrupt the integrity of intestinal epithelial tight junctions, contributing to a phenomenon commonly referred to as “leaky gut.” The LPS produced in the gut subsequently enters the systemic circulation through this compromised intestinal barrier and, aided by blood flow, binds to Toll-like receptor 4 (TLR4) on macrophages. This interaction activates the secretion of pro-inflammatory cytokines such as tumor necrosis factor-alpha (TNF-α) and interleukin-6 (IL-6), while also triggering the nuclear factor kappa-B (NF-κB) signaling pathway, thereby instigating a cascade of systemic chronic inflammation.

The BBB serves as a protective shield, preventing harmful substances such as LPS from directly entering the brain through the systemic circulation. Chronic inflammation induced by LPS indirectly affects the brain, with immune cells playing a central role in this process. Specifically, LPS triggers peripheral immune cells to release pro-inflammatory cytokines, which in turn compromise the integrity of the BBB (Banks and Erickson, 2010). These circulating cytokines subsequently reach the brain’s microcirculation, where they activate cytokines, chemokines, and free fatty acids. Elevated levels of free fatty acids activate TLR4 on microglia and astrocytes, initiating an inflammatory cascade. This cascade then stimulates neurons in the central nervous system (CNS), leading to neuroinflammation, BBB dysfunction, and the recruitment of peripheral immune cells into the CNS (Tucsek et al., 2013; Miller and Spencer, 2014).

Studies have demonstrated that intraperitoneal LPS injection in mice accelerates the accumulation of Aβ in the hippocampus, resulting in cognitive impairments (Friedland, 2015). Additional research indicates that LPS binds to microglial receptors, including TLR2, TLR4, and CD14, triggering a cytokine and chemokine cascade through myeloid differentiation factor 88 (MyD88) and NF-κB-dependent signaling pathways. This cascade disrupts neuronal homeostasis (Lukiw, 2016). Moreover, LPS activation of the NF-κB signaling pathway upregulates the expression of inflammatory mediators such as miRNA-146a and miRNA-155, further contributing to the progression of Alzheimer’s disease (Alexandrov et al., 2019).

The gut microbiota plays a crucial role in modulating peripheral immunity through its metabolites, which function as immune-active signaling molecules. SCFAs regulate the quantity and functionality of regulatory T cells (Tregs) by interacting with their receptor, G protein-coupled receptor 43 (GPR43) (Smith et al., 2013). Bile acids activate the farnesoid X receptor (FXR) and G protein-coupled bile acid receptor 1 (TGR5), leading to the elevated expression of these receptors in innate immune cells, including intestinal macrophages, dendritic cells, and natural killer T cells. This activation is pivotal in maintaining intestinal immune homeostasis (Biagioli et al., 2021). Indole derivatives, which are exclusively produced by the gut microbiota, influence the functional differentiation of CD4+ T cells into Tregs and T-helper 17 (Th17) cells (Roager and Licht, 2018). Furthermore, indole plays a key role in regulating the Tregs/Th17 balance in the mucosal environment, thereby modulating the anti-inflammatory and pro-inflammatory responses within various compartments of the gastrointestinal tract (Grifka-Walk et al., 2021).

#### 3.2.3 Regulatory effects of gut microbiota metabolites on AD

In comparison to cognitively healthy individuals, patients with mild cognitive impairment (MCI) and AD (Roager and Licht, 2018) AD dementia exhibit elevated cerebrospinal fluid (CSF) levels of TMAO. In absolute terms, CSF TMAO concentrations have been reported at sub- to low-micromolar levels (≈ 0.11–6.43 μM) in human studies (Rio et al., 2017), while Vogt et al. (2018) demonstrated higher CSF TMAO levels in MCI and AD using scaled intensity units without providing absolute values—limiting translation to clinical thresholds. Importantly, elevated CSF TMAO correlates with key AD pathological biomarkers, including phosphorylated tau, the p-tau/Aβ42 ratio, and neurodegeneration-associated proteins such as total tau and neurofilament light chain. Plasma studies similarly associate high circulating TMAO with accelerated Aβ deposition and cognitive decline, potentially mediated by enhanced β-secretase activity, disruption of neuronal signaling, and facilitation of platelet-derived Aβ entry into the brain (Zhu et al., 2016; Gao et al., 2019). Collectively, these findings suggest that sustained systemic TMAO elevation, particularly in individuals with cardiometabolic vulnerability, may predispose to AD-related neuropathology.

Mechanistic studies in cell and animal models provide deeper insights into how TMAO and its precursor trimethylamine (TMA) influence BBB integrity and AD-relevant pathways. In hCMEC/D3 endothelial monolayers, low-micromolar TMAO (≈4–40 μM) upregulates tight-junction proteins (e.g., claudin-1), reduces tracer permeability, and increases TEER (∼65%), thereby enhancing BBB integrity (Hoyles et al., 2021). Long-term TMAO treatment has also been shown to reduce astrocyte and microglial reactivity in specific brain regions, preserving cognitive function under inflammatory stress (Hoyles et al., 2021). In contrast, TMA, the precursor of TMAO, consistently impairs BBB function by disrupting tight-junction integrity and increasing permeability (Hoyles et al., 2021). However, chronic elevation of TMAO in AD mouse models promotes tau conformational changes and aggregation (Tseng and Graves, 1998), increases hippocampal β-secretase activity and amyloid-β deposition (Gao et al., 2019), activates the PERK–EIF2α–ER stress pathway (Govindarajulu et al., 2020), impairs hippocampal synaptic plasticity via the mTOR/P70S6K/4EBP1 axis (Li et al., 2018; Zhou et al., 2023), and upregulates circulating clusterin, potentially driving neuroinflammation and β-secretase–dependent Aβ pathology (Yu et al., 2013). In vascular dementia models, elevated TMAO further exacerbates cognitive deficits and neuropathological changes by inhibiting the SIRT1 pathway and inducing oxidative stress–mediated apoptosis (Deng et al., 2022).

Taken together, the effects of the TMA/TMAO axis on AD pathophysiology are dose-, duration-, baseline-, and model-dependent rather than uniformly harmful or beneficial. Human data remain largely associational, showing that higher CSF or plasma TMAO correlates with biomarker-defined disease burden and cognitive decline, especially in metabolic or vascular vulnerability. Preclinical studies provide causal mechanistic evidence by demonstrating that excessive or chronic TMAO exposure can worsen tau, Aβ, synaptic, and inflammatory pathways, whereas physiological-range, short-term TMAO may stabilize BBB function and offer transient neuroprotection under inflammatory stress. TMA, in contrast, appears consistently detrimental to BBB integrity. Overall, modulating the TMA/TMAO metabolic axis may represent a promising therapeutic target, but its clinical translation requires careful phenotyping, exposure control, and integration of baseline cardiometabolic status. Given that human studies are currently correlational, well-designed longitudinal cohorts and interventional trials are essential to clarify causality.

**Table:** Differences and effects of TMA and TMAO administration.

Substance	Model	Administration method	Dose	Main effects
TMA	hCMEC/D3 cells (human brain microvascular endothelial cells)	Transwell system	0–40 μM	Disrupts tight junctions, increases BBB permeability.
TMAO	hCMEC/D3 cells (human brain microvascular endothelial cells)	Transwell system	4–40 μM (low); 4 mM (high)	At 4–40 μM: upregulates tight junction proteins (claudin-5, occludin), increases TEER (∼65%), reduces permeability; decreases TNFα-induced U937 adhesion. At 4 mM: protective effect lost, permeability increased.
TMAO	Mice	Intraperitoneal injection (ip)	1.8 mg/kg	Reduces Evans Blue leakage (2 h). Attenuates LPS-induced BBB disruption. Alters brain transcription (mitochondrial pathways). Prevents memory impairment and BBB breakdown in chronic inflammation.
TMAO	Mice	Drinking water administration	0.5 mg/ml (long-term)	Prevents LPS-induced IgG deposition around cerebral vessels. Reduces GFAP+ astrocytic and Iba1+ microglial reactivity in hippocampus and entorhinal cortex, preserving cognition.

This table is based on the study: Regulation of blood–brain barrier integrity by microbiome-associated methylamines and cognition by trimethylamine N-oxide (Hoyles et al., 2021).

SCFAs are key metabolic products of the gut microbiota and have been shown to mitigate cognitive impairments associated with AD and other conditions, including isoflurane exposure, scopolamine administration, and radiation. Butyrate, in particular, has been found to upregulate the expression of phosphorylated cAMP response element-binding protein (CREB) and BDNF in the hippocampus, thereby improving cognitive dysfunction induced by radiation (Lee et al., 2019). Furthermore, butyrate enhances associative memory in APPPS1-21 mice and reduces early-stage neuroinflammation in 5xFAD mice, contributing to improved synaptic plasticity (Govindarajan et al., 2011; Jiang et al., 2021). In microglial cells, butyrate activates the PI3K/AKT/CREB/BDNF signaling pathway, further promoting synaptic plasticity (Saw et al., 2019).

Metabolites, including SCFAs, have shown potential in alleviating symptoms of AD by reducing Aβ deposition and tau hyperphosphorylation, inhibiting neuroinflammation, improving blood-brain barrier integrity, and regulating neuroendocrine functions. SCFAs play a significant role in modulating both Aβ and tau pathologies. In murine models, butyrate activates nicotinic acid receptor 1, downregulates amyloid precursor protein expression, and upregulates AD-related proteins such as nephronectin and BDNF, effectively reversing Aβ-induced damage in neuroblastoma cells (Sun et al., 2020). Propionate has been shown to reduce Aβ-induced neurotoxicity in human neuroblastoma SH-SY5Y cells by inhibiting NF-κB signaling and decreasing the secretion of cyclooxygenase-2 and inducible nitric oxide synthase (Filippone et al., 2020). Furthermore, butyrate mitigates tau hyperphosphorylation and reduces the expression of inflammation-related proteins, such as glial fibrillary acidic protein, by inhibiting histone acetylation, thereby alleviating AD pathology (Cao et al., 2018).

Neuroinflammation, mediated by microglia and astrocytes as components of the central innate immune system, plays a key role in immune homeostasis and synaptic plasticity in the central nervous system. Acetate has been shown to reduce neuroinflammation by downregulating the pro-inflammatory cytokine interleukin-1β, inhibiting microglial activation, and decreasing inflammation induced by LPS (Soliman et al., 2012b). Additionally, acetate exerts anti-inflammatory effects by upregulating GPR41 expression, suppressing the ERK/JNK/NF-κB signaling pathways, and lowering COX-2 and IL-1β levels (Liu et al., 2020). Butyrate modulates signaling through protein kinase B-small GTPase pathways by inhibiting histone deacetylase activity, reducing microglial process elongation, and alleviating neuroinflammation (Li et al., 2017).

Astrocytes are crucial in maintaining central nervous system homeostasis by regulating neurotransmitter levels, synaptic plasticity, and blood-brain barrier integrity (Verkhratsky and Nedergaard, 2018). Acetate exerts its effects by mediating histone deacetylation, inhibiting MAPK and NF-κB signaling pathways, and reducing pro-inflammatory cytokines such as IL-1β, MCP-1, TNF-α, and IL-6. Additionally, acetate upregulates TGF-β1 signaling, promoting anti-inflammatory cytokine IL-4 production, thereby mitigating neuroinflammation induced by astrocyte activation (Soliman et al., 2012a). Furthermore, acetate alleviates neuroinflammation by inhibiting phospholipase A2, cPLA2-IIA, and phospholipase Cβ1 signaling in primary astrocytes through histone acetylation (Soliman et al., 2013).

SCFAs can mitigate AD by improving blood-brain barrier (BBB) dysfunction. BBB dysfunction and cerebrovascular lesions are frequently associated with AD pathological markers such as Aβ deposition and tau hyperphosphorylation. This dysfunction may initiate a vicious cycle, where brain Aβ accumulation exacerbates cerebrovascular damage during AD progression (Yamazaki and Kanekiyo, 2017). SCFAs stimulate the NLRP6 inflammasome, improving intestinal epithelial barrier integrity, which in turn prevents hippocampal neuroinflammation and neuronal loss induced by a high-fructose diet (Li et al., 2019). Butyrate, for instance, upregulates the expression of occludin and ZO-1 in the brain, thereby repairing BBB damage and improving neurofunctional deficits (Li et al., 2016). Furthermore, valproic acid-mediated histone deacetylase inhibition suppresses NF-κB expression, downregulates matrix metalloproteinase-9 levels, and reduces the degradation of tight junction proteins, facilitating BBB repair in rats with transient focal cerebral ischemia (Wang et al., 2010).

However, the precise mechanisms by which SCFAs regulate microglial transcriptomics and functional activities remain unclear. Future studies should employ integrated approaches, including metagenomics, transcriptomics, and proteomics, to elucidate the role and mechanisms of gut microbiota metabolites in influencing AD pathology.

The gut microbiota plays a crucial role in the biotransformation of bile acids (BAs), thereby regulating the composition and homeostasis of the BA pool, which in turn influences its physiological functions (Ridlon et al., 2014). Current research on the neuroprotective effects of BAs has primarily focused on tauroursodeoxycholic acid (TUDCA). Studies have demonstrated that six months of TUDCA supplementation can reduce Aβ deposition in the hippocampus and prefrontal cortex of AD model mice, improving their spatial recognition and memory deficits (Lo et al., 2013). Additionally, TUDCA alleviates endoplasmic reticulum stress by restoring the levels of IRE1α, pPERK, and BIP proteins, thereby reducing excessive Tau phosphorylation (van der Harg et al., 2014). Further investigations have revealed that TUDCA enhances AKT signaling and suppresses glycogen synthase kinase 3β (GSK-3β) expression, leading to the attenuation of astrocyte and microglial hyperactivation in APP/PS1 mice, which in turn reduces neuroinflammation, Tau phosphorylation, and Aβ accumulation (Dionísio et al., 2015). Moreover, TUDCA has been shown to inhibit Aβ-induced apoptosis in PC12 cells by downregulating the E2F-1/p53/Bax pathway (Ramalho et al., 2004).

Furthermore, middle-aged hypercholesterolemia is considered a significant risk factor for AD. Studies suggest that elevated cholesterol levels can be transported to neurons via astrocyte-secreted apolipoprotein E (ApoE), thereby influencing β/γ-secretase-mediated amyloid precursor protein (APP) processing in perineuronal regions, ultimately promoting Aβ production (Wang H. et al., 2021). Thus, modulating gut microbiota to enhance cholesterol metabolism and facilitate the production of neuroprotective BAs may represent a potential strategy for mitigating AD progression (Shulpekova et al., 2022).

## 4 Exercise and gut microbiota

At the exercise modality level, various forms of exercise were associated with changes in the gut microbiota, which were linked to gut barrier integrity and lower inflammatory responses. Some studies were conducted in non-AD backgrounds, including Parkinson’s disease, obesity, gastrointestinal disorders, postoperative cognitive dysfunction (POCD), and experimental autoimmune encephalomyelitis (EAE) models. These findings were included to illustrate the broader effects of exercise on gut microbiota and cognition but should be interpreted cautiously when extrapolating to AD pathology. An 8-week aerobic exercise intervention in elderly women with limited physical activity revealed an increase in taxa associated with anti-inflammatory functions, such as Verrucomicrobia, and a decrease in taxa associated with pro-inflammatory activity, such as Proteobacteria (Zhong et al., 2020). Similarly, a 6-month randomized controlled trial by Kern et al. (2019) involving 88 obese participants showed that high-intensity anaerobic exercise was associated with greater gut microbiota diversity. Furthermore, Zheng et al. (2020) demonstrated that Tai Chi enhanced both the abundance and diversity of beneficial bacteria across different age groups, while simultaneously reducing the expression of immune regulators, serum inflammatory factors, and TNF-α. On the other hand, a study by Taniguchi et al. (2018), conducted in non-AD elderly men over a 5-week aerobic exercise randomized crossover trial, observed a significant decrease in the relative abundance of *Clostridioides difficile* and an increase in *Oscillospira*, but these changes were not associated with the aerobic exercise intervention. This suggested that aerobic exercise did not affect gut microbiota diversity and composition. These discrepancies could be due to factors like exercise type, frequency, and duration.

Matsumoto et al. (2008) found that 5 weeks of voluntary wheel running could influence changes in the gut microbiota composition of mice, increasing the levels of butyrate, a metabolite of the gut microbiota, and subsequently improving gastrointestinal diseases in rats (non-AD background). Queipo-Ortuño et al. (2013) found that 6 days of voluntary wheel running significantly upregulated the relative abundance of microbial taxa with putative modulatory roles, such as lactic acid bacteria, *Bifidobacterium longum*, and *Bacillus sphaericus*. Fernández et al. (2021) conducted a 6-week (5 days/week) resistance combined endurance exercise intervention in 26 mice and found that the relative abundance of *Ruminococcus gnavus* significantly decreased, while the abundance of *Parabacteroides* increased. Chen et al. (2021) showed that anaerobic resistance exercise could increase the gut microbiota abundance and diversity in experimental autoimmune encephalomyelitis mice (non-AD background), reduce the Firmicutes/Bacteroidetes ratio, and decrease intestinal mucosal permeability.

Additionally, this exercise enhanced regulatory T-cell responses by influencing the expression of helper T-cell 17, thereby improving neurological diseases. Plissonneau et al. (2021) found that 12 weeks of anaerobic training in obese rats significantly reduced adipocyte size in subcutaneous and visceral adipose tissue, while modulating gut-microbiota α- and β-diversity and increasing the relative abundance of Tenericutes and Proteobacteria.

In contrast, Maillard et al. (2019) found that high-intensity anaerobic exercise could suppress inflammatory responses and regulate metabolic disorders of glucose and lipids, but these changes were not related to alterations in the composition of the gut microbiota. The above studies suggest that exercise can influence the diversity of the gut microbiota, significantly regulate its composition, and suppress the body’s inflammatory responses. However, there is heterogeneity in the impact of exercise on the gut microbiota, with most studies confirming that exercise increases gut microbiota diversity and abundance, preventing intestinal damage. On the other hand, some studies have found that the beneficial effects of exercise on the body are not related to changes in the overall composition and structure of the gut microbiota. The differences in the results of studies on exercise and gut microbiota may be related to factors such as the study subjects, methods, exercise intensity, exercise load, and exercise modalities, and further in-depth research is needed. However, existing literature lacks specific descriptions of exercise form, intensity, duration, and volume, making it difficult to summarize the effects of more complex exercise forms/strengths and durations on the gut microbiota. Additionally, no studies have explored the impact of exercise interventions on the gut microbiota from the perspective of individual differences in the participants (Tables 1a, b).

**TABLE 1a:** (Human): Effects of different exercise modalities on gut microbiota.

Type and intensity of exercise	Population/model	Outcome	References
8 weeks of Combined aerobic and resistance training, 4 times per week, 60 min per session	Elderly women (human)	Gut microbiota diversity-Verrucomicrobia↑, Proteobacteria↓ At the phylum, class, and order levels, significant differences were found between the exercise and control groups in Fusobacteria, Betaproteobacteria, and Bifidobacteriales. A significant interaction between the two groups was observed for Actinobacteria. At the family and genus levels, exercise significantly affected the abundance of *Bifidobacteriaceae*, *Bifidobacterium*, and *Gemmiger*.	Zhong et al., 2020
A 6-month intervention with exercise 5 days per week, at an intensity of either 50% or 70% of VO_2_ peak reserve, corresponding to a weekly energy expenditure of 1,600 kcal for women and 2,100 kcal for men, performed either as leisure-time exercise or active commuting by bike.	Obese population (human)	Gut microbiota diversity↑ Increased by 5% in the VIG (vigorous intensity exercise) group at 3 months compared to the CON group. No associations were found between alpha diversity and phenotypical outcomes. Significant changes in beta diversity were observed in all exercise groups compared to the CON group, with decreased heterogeneity in the VIG group. The inferred functional potential of the microbiota increased in the exercise groups, particularly in the MOD group and at 3 months.	Kern et al., 2019
Intervention lasted 12 weeks; S group: Tai Chi, 30 min/session, 5 times/week; L group: Tai Chi, 60 min/session, 5 times/week; Control group: jogging, 30 min/session, 5 times/week.	Patients with gastrointestinal disorders (human)	*Bifidobacterium*↑ *Bacteroides* rectus↓ *Enterobacter*↓	Zheng et al., 2020
Participants completed a supervised, progressive 5-week aerobic exercise program consisting of three cycle ergometer sessions per week, with intensity increased from 60% VO_2_ peak (30 min, week 1) to 70% VO_2_ peak (30 min, weeks 2–3) and 75% VO_2_ peak (45 min, weeks 4–5).	Elderly men (human)	*Clostridioides difficile*↓ *Oscillospira*↑ limited impact on gut microbiota diversity and composition; Changes in α-diversity indices during the intervention were negatively correlated with changes in systolic and diastolic blood pressure, especially during exercise;	Taniguchi et al., 2018

**TABLE 1b:** (Animal): Effects of different exercise modalities on gut microbiota.

Exercise type	Type and intensity of exercise	Population/ model	Research results	References
Running	6 days of self-selected voluntary wheel running with aerobic, continuous or intermittent patterns	Male rat (animal)	*Lactobacillus*, *Bifidobacterium*, B. coccoides-E. ↑ A positive correlation between *Bifidobacterium* and *Lactobacillus* and serum leptin levels; A negative correlation between Clostridium, *Bacteroides*, *Prevotella*, and serum leptin levels; *Bifidobacterium*, *Lactobacillus*, and B. coccoides-*Eubacterium* rectale group showed a negative correlation with serum ghrelin levels; *Bacteroides* and *Prevotella* showed a positive correlation with serum ghrelin levels;	Queipo-Ortuño et al., 2013
Treadmill and vertical ladder	Endurance training: 4 weeks of moderate-to-high intensity treadmill running (40%–80% of maximal speed), 60 min/session, 5 sessions/week, with a fixed distance of 1,000 m per session. Resistance training: 4 weeks of tail-weighted ladder climbing (25%–65% of maximal load), 400–2,000 steps/session, with a fixed work of 260 mJ per session, 5 sessions/week.	Mice (animal)	RES (Resistance) →Clostridium and C. cocleatum, *Alistipes*↑, *Desulfovibrio* sp. ↓ Trained mice →*Ruminococcus gnavus*, ↑ *Parabacteroides* (vs. CTL) *Ruminococcus gnavus*↓, genus *Parabacteroides*→ END (Endurance) ↑Microbiota diversity and evenness↑, *Desulfovibrio*, *Desulfovibrio* sp. *Prevotellaceae*, *Prevotella*, *Akkermansia muciniphila*↑, Lachnospiraceae, Lactobacillaceae↓	Fernández et al., 2021
Stair climbing	Strength training was conducted using a mouse wheel fatigue tester, 6 days per week for 4 weeks, with session durations of 20, 40, or 60 min depending on group assignment	Multiple sclerosis (MS) Mice (animal)	Microbiota diversity↑ Firmicutes/Bacteroidetes ↓ Treg/Th17↑ disease severity and neuropathology↓	Chen et al., 2021
Running	Exercise training consisted of treadmill running at 0° inclination, 5 days/week for 10 weeks, with HIIT rats performing 6 × 4-min bouts at 18 m ⋅min^−1^ interspersed with 3-min at 10 m ⋅ min^−1^, and MICT rats running continuously at 12 m ⋅min^−1^ (equalized total distance).	Obese male Zucker rats (animal)	HIIT→inflammation↓, Zonula occludens-1 and occludin expression↑ HIIT and MICT→glucose metabolism↑, plasma LBP↓ No changes in gut microbiota composition in both HIIT and MICT groups.	Maillard et al., 2019

## 5 Exercise and Alzheimer’s disease

### 5.1 The impact of exercise on Alzheimer’s disease

Regular physical activity has been consistently associated with a lower incidence and slower progression of AD. Erickson et al. (2011) reported that a 6-month moderate-intensity aerobic intervention in healthy elderly individuals induced structural changes in the prefrontal cortex and increased hippocampal volume, resulting in improved spatial memory. Interestingly, regions such as the hypothalamus and caudate nucleus were unaffected, suggesting that exercise influences region-specific molecular pathways rather than exerting uniform effects across the brain. Aerobic exercise or resistance training can improve agility, balance, and flexibility in elderly AD patients (Garuffi et al., 2012; Hong et al., 2017; Northey et al., 2017). Further research has found that the neurophysiological mechanisms by which exercise improves AD are related to the upregulation of BDNF secretion and its signaling pathways, as well as a reduction in Aβ deposition and tau phosphorylation. Rosa et al. (2019) conducted a long-term exercise intervention in 86 healthy middle-aged men and found that exercise directly activates neuroprotective and neurotrophic signaling pathways downstream of BDNF, modulating peripheral levels of BDNF and tissue plasminogen activator, thus delaying physiological memory loss and brain degeneration. In a recent study on double transgenic mice, voluntary wheel running was found to reduce Aβ deposition and tau hyperphosphorylation in the hippocampus, as well as astrocyte proliferation, which subsequently improved spatial memory and alleviated AD pathological features (Tapia-Rojas et al., 2015). Building upon this, Um et al. (2011) further investigated and found that 12 weeks of wheel running reduced p38MAPK and tau (Ser404, Ser202, Thr231) phosphorylation levels, inhibited Bax expression, and increased PI3K, Akt, GSK-3α/β phosphorylation levels, NGF, BDNF, and hippocampal Bcl-2 expression, resulting in decreased serum levels of TC, insulin, glucose, and corticosterone. This study suggests that exercise may inhibit neuronal cell death in AD transgenic mice, thereby preventing and treating AD. Additionally, Leem et al. found that three months of aerobic exercise upregulated the expression of β-catenin at the cytoplasmic and nuclear levels, as well as T cell factor-4 (Tcf-4) and cyclin D1 in the brain. This change led to a significant reduction in the level of phosphorylated tau protein in the CA3 region of the hippocampus in mice post-exercise. This study suggests that long-term aerobic exercise has been associated with alleviation of tau pathology (Leem et al., 2009). Moreover, long-term wheel running decreases tau phosphorylation levels through the PI3K-Akt pathway, reduces glycogen synthase kinase-3β activity, ^⋅^and increases BDNF levels in the central nervous system, thereby enhancing neuronal survival and differentiation (Ohia-Nwoko et al., 2014). Singh et al. (2014) conducted a randomized double-blind controlled trial on adults with mild cognitive impairment, and found that resistance training improved overall cognitive and executive function, with effects maintained during an 18-month follow-up. Another study found that in a six-month aerobic exercise intervention, changes in executive control, selective attention, processing speed, and cognitive flexibility were more pronounced in women than in men. The gender differences in cognitive response may be related to the metabolic effects of exercise, with mechanisms potentially involving improved cortisol, glucose regulation, and insulin sensitivity in women, whereas these effects were not observed in men. BDNF is highly regulated by the HPA axis activity in women (Baker et al., 2010).

Twelve weeks of moderate-intensity wheel running not only enhanced the ability of AD mice to transport Aβ from the brain to the periphery but also increased the expression of LRP-1 protein in the liver (Xiong et al., 2015). The 12-week wheel running significantly reduced the conversion of pro-inflammatory M1-type microglia to anti-inflammatory M2-type microglia in APP/PS1 mice, decreased the proportion of M1-type microglia, and suppressed brain neuroinflammation. Furthermore, wheel running also regulated oxidative stress levels by reducing malondialdehyde levels, increased the activity of SOD and Mn-SOD in the hippocampus, effectively preventing Aβ deposition and exacerbation of cognitive dysfunction in AD mice (Zhang et al., 2019).

AD, as a highly complex neurological disease, currently lacks a definitive cure. Both animal and clinical studies have demonstrated that exercise can improve cognitive and memory dysfunction in AD. On one hand, from the perspective of exercise itself, to further enhance neural plasticity in the field of exercise cognition and improve cognitive functions related to exercise execution and control, training programs emphasizing coordination and simple operations should be explored. On the other hand, from the standpoint of lifestyle interventions, multimodal interventions combining exercise, nutritional strategies, cognitive interventions, and physical stimulation should be considered as future research directions, aiming to unlock the maximum potential of combined approaches in AD treatment. It is believed that these studies will play a key role in the prevention and treatment of AD in the future (Tables 2a, b).

**TABLE 2a:** (Human): Effects of different exercise modalities on Alzheimer’s disease.

Type and intensity of exercise	Population/model	Research results	References
6 months of supervised walking, 3–5 times per week, 40 min per session (progressively increased from 10 min), at 50%–60% HRR initially and 60%–75% HRR thereafter.	Elderly (human)	Increased anterior hippocampus volume by 2%, reversing age-related volume loss by 1 to 2 years. Improved spatial memory. Increased hippocampal volume was associated with higher serum BDNF levels	Erickson et al., 2011
The resistance training intervention lasted 16 weeks, with sessions performed 3 times per week for ∼60 min each, at ∼85% of 20RM, consisting of machine- and free-weight exercises targeting five major muscle groups.	AD patients (human)	Significant improvements Daily Living Performance (Moving around the house, climbing stairs, standing up from the floor, putting on socks); Improved agility, lower limb strength, balance, and flexibility in AD patients.	Garuffi et al., 2012
Athletes with long-term training history (average training duration: 35 ± 15 years).	Healthy middle-aged men (human)	Significant improvement in memory performance in the Free and Cued Immediate Recall tests; Significant decrease in plasma malondialdehyde (a marker of lipid peroxidation); Significant reduction in serum BDNF and plasma Cathepsin B (CTSB) levels, which were inversely correlated with weekly hours of exercise;	Rosa et al., 2019
A 6-month supervised intervention, 2–3 sessions/week; Progressive Resistance Training (PRT): 5–6 major muscle groups, 3 × 8 reps each, high-intensity pneumatic resistance machines;	Adults with mild cognitive impairment (human)	Significant improvement in ADAS-Cog scores; Significant improvement in executive function (Wechsler Adult Intelligence Scale Matrices), with this improvement sustained over 18 months	Singh et al., 2014
A 6-month supervised program, 4 sessions/week for 45–60 min; the aerobic group exercised at 75%–85% HRR after a 6-week progression (treadmill, bike, elliptical), while the control group performed stretching and balance exercises at ≤ 50% HRR.	Elderly with mild cognitive impairment (human)	Cognition:Women: Improved performance on multiple executive function tests. Men: Beneficial effect only on Trails B performance. Glucose Metabolism:Women: Improved glucose disposal, reduced fasting plasma insulin and cortisol levels. Men: No significant effects on glucose metabolism. Trophic Activity:Women: Decreased brain-derived neurotrophic factor (BDNF) levels. Men: Increased insulin-like growth factor I (IGF-I) levels.	Baker et al., 2010
An 8-week program with 3 sessions/week, 60 min/session; the exercise group performed moderate-intensity aerobic training (65%–75% HRmax, RPE 5–6; treadmill, cycle ergometer, elliptical), including 10 min warm-up, 40 min exercise, and 10 min cool-down	MA-dependent individuals	Significantly improved working memory and inhibition ability. Significantly increased levels of *Bifidobacterium*, *Lactococcus lactis*, *Prevotellaceae*, and *Ruminococcaceae*. Significantly decreased levels of *Desulfovibrio* and *Akkermansia*. Cognitive function was positively correlated with *Bifidobacterium*, *Dialister*, and *Adlercreutzia*, and negatively correlated with *Enterobacteria*, *Bacillus cereus*, *Catabacter*, and *Akkermansia*.	Zhu et al., 2023

**TABLE 2b:** (Animal): Effects of different exercise modalities on Alzheimer’s disease.

Type and intensity of exercise	Study subjects	Research results	References
A 12-week treadmill program, 5 days/week, 60 min/day, at 12 m/min on a 0% gradient.	Tg mice (animal)	Improved cognitive function in the water maze test. Decreased expression of Aβ-42, Cox-2, and caspase-3. Increased expression of NGF, BDNF, and phospho-CREB. Aβ-dependent neuronal cell death was significantly suppressed in the hippocampus.	Um et al., 2011
A 12-week treadmill program, 5 days/week, 60 min/day; low intensity (12 m/min, ∼50%–60% VO_2_max) or high intensity (19 m/min, ∼70%–80% VO_2_max).	Tau protein Tg mice (animal)	Increased the expression and enzymatic activities of Cu/Zn-superoxide dismutase (SOD) and catalase in the brain. Significantly reduced the levels of phosphorylated tau in the brains of Tg mice.	Leem et al., 2009
A 12-week forced treadmill program, 5 days/week, 40 min/day, with progressive speeds up to 15 m/min (total ∼450 m/day, ∼27 km over the intervention), performed at 0° inclination.	Mice (animal)	Improved overall locomotor ability and exploratory activity in P301S tau mice. Significantly reduced full-length tau and hyperphosphorylated tau levels in the spinal cord and hippocampus. Failed to attenuate significant neuronal loss in the hippocampus and cortex.	Ohia-Nwoko et al., 2014
A 5-month treadmill training program, 6 days/week, with daily running distance progressively increased from 70 m at 5–8 m/min to 300 m at 10–15 m/min, thereafter maintained, corresponding to ∼30%–40% of VO_2_max.	AD mice (animal)	Significantly improved spatial memory ability; Increased the number of BDNF-positive cells in the cerebral cortex and hippocampus; Reduced the ratio of activated microglia (Iba-1 positive) in the cerebral cortex and hippocampus; No significant effect on β-amyloid deposition in the cerebral cortex and hippocampus	Xiong et al., 2015
12 weeks of training, 5 days per week, 45 min per session, at an intensity of 5–12 m/min.	APP/PS1 mice (animal)	Significantly reduces the proportion of M1 microglia and increases the activity of SOD and Mn-SOD in the hippocampus	Zhang et al., 2019
20-week aerobic treadmill training, starting at 12 m/min for 10 min and progressively increasing to 15 m/min for 20 min, one session per day, five days per week.	APP/PS1 and C57BL/6 mice (animal)	Delayed cognitive decline. Relative abundance of Bacteroidetes significantly decreased. *Ileibacterium* and *Faecalibaculum* significantly increased. Lipid metabolism and bile acid metabolism pathways were significantly enriched in the cecal microbiota.	Wei et al., 2025
Aerobic treadmill exercise was performed for 12 weeks, 5 days per week, 60 min per session, at a constant speed of 12 m/min and 0% incline.	APP/PS1 mice	Cognitive function: Improvement in memory and learning abilities in young APP/PS1 mice. Tau Phosphorylation: Reduction of tau phosphorylation in young mice. Neuroinflammation and synaptic plasticity: Changes in neuroinflammation and synaptic plasticity markers in young mice. Metabolites: L-Valine, Glucosamine, Formylanthranilic acid, and Myristic acid in circulation. Gut microbiota diversity: Changes in microbial populations like *Oscillibacter*, *Alistipes*, and others. Biomarkers: Reduction in ADAM10 and GFAP expression in middle-aged mice.	Wang G. et al., 2021

### 5.2 Heterogeneity in exercise effects on AD

While exercise benefits Alzheimer’s disease (AD) through multiple pathways—including structural remodeling, molecular signaling, metabolic regulation, and gut–brain axis modulation—effects are not uniform and are shaped by participant characteristics, disease stage, exercise parameters, and methodological factors. Across available trials, benefits are generally larger in individuals with MCI than in those with established dementia, and appear more consistent in younger or “younger-old” adults than in the oldest-old.

RCTs show that resistance training significantly improves executive function in individuals with MCI, whereas aerobic exercise more strongly supports hippocampal plasticity and cognitive flexibility, with women exhibiting greater gains (Baker et al., 2010; Erickson et al., 2011; Singh et al., 2014). Emerging evidence highlights that high-intensity interval training (HIIT) and mind–body practices such as Tai Chi may exert distinct benefits by modulating systemic inflammation and gut microbiota composition, suggesting novel intervention pathways. Additionally, intervention outcomes may depend on exercise frequency, duration, and intensity, underscoring the need for standardized dosing in future studies.

Mechanistically, these heterogeneous responses may arise from differential activation of biological pathways: aerobic exercise preferentially enhances BDNF-dependent signaling and hippocampal neurogenesis, resistance training modulates glucose homeostasis and executive control networks, and sex-specific differences may relate to HPA-axis regulation and metabolic adaptations.

Interpretation requires caution because diet, body weight, sleep, and medication use were rarely controlled, especially in cross-sectional studies. Future research should prioritize sex-balanced cohorts, adopt stratified analyses, and leverage longitudinal designs and multi-omics profiling to clarify causal pathways and optimize personalized exercise-based interventions for AD.

## 6 Exercise regulates gut microbiota to influence Alzheimer’s disease

Exercise has been associated with improvements in AD, potentially linked to modulation of gut microbiota and neuroendocrine pathways. Exercise induces an increase in the abundance of *Faecalibaculum* and *Ileibacterium* in AD mice, affecting lipid and bile acid metabolism, thereby improving cognitive impairment and slowing the pathological progression of AD in these mice (Wei et al., 2025). A 16-week voluntary wheel running study on young and middle-aged mice found that exercise improved cognitive function in young mice but had no effect on middle-aged APP/PS1 mice. This difference may be due to the mechanism in young mice, where exercise reduces tau protein hyperphosphorylation by inhibiting p-GSK3β activity. This discrepancy might be related to the mechanism where voluntary exercise in young mice reduces tau protein hyperphosphorylation by inhibiting p-GSK3β activity. Additionally, voluntary exercise increased the levels of L-valine, amino-glucose, formylated p-aminobenzoic acid, and myristic acid in the circulation, upregulated the abundance of *Oscillibacter*, and decreased the abundance of *Alistipes*, improving gut microbiota diversity and enhancing learning and memory ability in young mice after exercise. The study also found that, for middle-aged mice, exercise only reduced the expression of ADAM10 and GFAP proteins in the hippocampus, but no significant changes were observed in circulating metabolites. Therefore, starting exercise training at an earlier stage may have a stronger beneficial effect on cognitive and memory abilities, delaying the onset of neurodegenerative diseases such as AD (Wang G. et al., 2021). Aerobic exercise can upregulate the abundance of bacteria such as *Lactobacillus*, *Lactococcus lactis*, *Prevotellaceae*, and *Ruminococcaceae*, while modulating the abundance of bacteria such as *Desulfovibrio* and *Akkermansia*, whose effects may vary depending on host background, diet, and disease state (Zhu et al., 2023).

Exercise has been associated with alterations in gut microbiota composition, which correlate with suppressed neuroinflammation, reduced Aβ deposition, regulated tau phosphorylation, and preserved blood-brain barrier integrity. Probiotic and exercise combined interventions can reduce Aβ protein deposition in the hippocampus of transgenic mice, suppress neuroinflammatory responses, and play a role in improving memory deficits and inhibiting AD pathological mechanisms (Abraham et al., 2019). A 6-week wheel running exercise can reshape the gut microbiota in mice, inhibit neuroinflammation, reduce Aβ brain deposition, and improve cognitive and memory dysfunction (Kang et al., 2014). A 12-week voluntary wheel running intervention in male mice increased the relative abundance of *Lactobacillus* and altered the abundance of Clostridia_UCG-014 and *Akkermansia*, with potential functional implications dependent on experimental context (Li et al., 2023). Aerobic exercise interventions can regulate the gut microbiota α-diversity in rats with post-operative cognitive dysfunction (non-AD background), increase the relative abundance of Firmicutes, and decrease the relative abundance of Bacteroidetes, maintaining the balance of gut microbiota structure and suppressing neuroinflammation, thereby improving cognitive memory abilities in rats (Feng et al., 2017). Chronic exercise can also increase the abundance of *Akkermansia muciniphila*, *Muribaculum* and *Acidaminococcus*, while decreasing the abundance of *Bacteroides*, *Clostridium perfringens*, *Bacillus subtilis*, and *Helicobacter*. At the same time, it upregulates the expression of tight junction proteins in the blood-brain barrier, reduces blood-brain barrier permeability, inhibits neuroinflammation, and improves brain function and cognitive behavior (Li et al., 2017; Jin et al., 2023). However, these findings should be interpreted with caution, as potential confounding factors such as dietary habits, body weight changes, sleep quality, and medication use were not consistently controlled across studies.

Kolonics et al. (2023) conducted a study on AD mouse models with physical exercise combined with supplementation of *Bifidobacterium longum* and *Lactobacillus* acidophilus. They found that this intervention improved the antioxidant system by activating LONP1, reduced the expression of NF-kB-related genes, decreased Aβ-40 aggregate accumulation, and reduced neurotoxicity. This intervention inhibited inflammation while maintaining mitochondrial homeostasis.

Abraham et al. (2019) demonstrated that exercise combined with probiotics could reduce Aβ deposition, activate microglia, and reduce OGG-1 accumulation in AD mice, thereby better preserving DNA integrity. Their study also showed that exercise or probiotics alone could not effectively improve spatial memory. However, the combined effect of these treatments enhanced brain performance in AD mice, with some of the beneficial effects mediated by changes in the gut microbiota. This suggests that exercise and probiotic-induced microbiome modulation can delay the pathological progression of AD and reduce its incidence.

**FIGURE 1:**
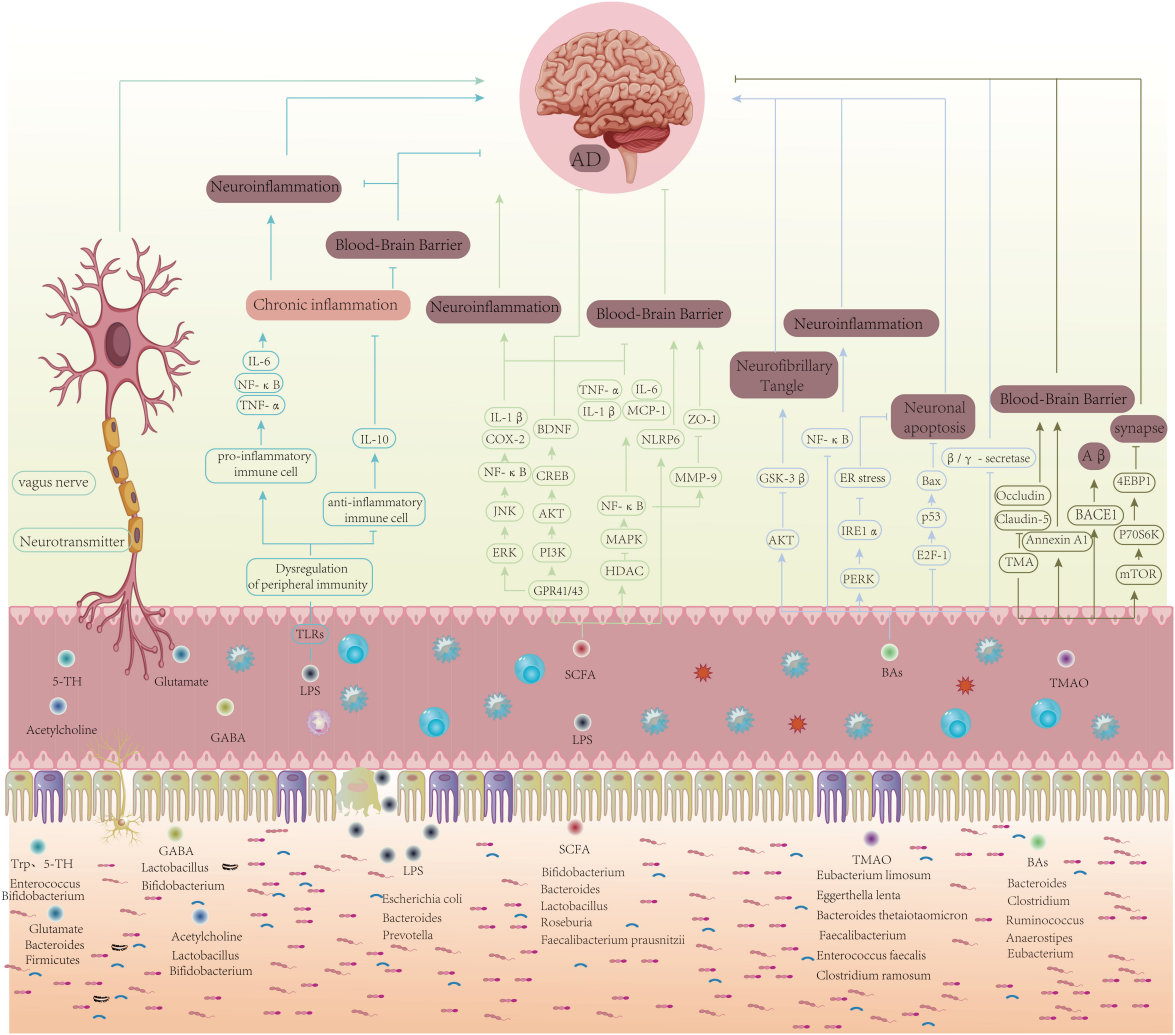
Schematic of how exercise may modulate AD pathology via the gut–brain axis: gut microbiota and metabolites, immune–inflammatory signaling, neurotransmitters/vagus nerve, and BBB integrity converge to influence Aβ/tau dynamics, synaptic plasticity, and neuronal survival.

Exercise can suppress neuroinflammation by regulating the levels of gut microbiota metabolites, such as SCFAs. Studies have shown that 5 weeks of wheel running can regulate the gut microbiota diversity in postoperative cognitive dysfunction mice, suppress neuroinflammation, and improve cognitive memory function (Moller et al., 1998). Abraham et al. (2019) established an AD mouse model and found that a combination of probiotics and aerobic exercise increased the abundance of butyrate-producing bacteria and reduced Aβ deposition in the hippocampus, thus slowing the progression of AD. Six weeks of voluntary wheel running effectively increased the butyrate/acetate ratio in the gut of mice, altered the gut microbiota composition, and reduced neuroinflammation (Allen et al., 2017). Another study found that excessive intake of TMAO (trimethylamine N-oxide) disrupts BBB permeability, leading to gut dysbiosis, which activates astrocytes and increases tau phosphorylation, thus accelerating cognitive impairment. Voluntary exercise intervention can reverse TMAO-induced cognitive dysfunction by reducing circulating TMAO and its precursors, increasing gut microbiota α-diversity, reshaping the gut microbiota balance, and regulating tau phosphorylation to maintain gut-brain integrity, ultimately improving the onset of AD (Zhang et al., 2023) (Figure 1).

## 7 Discussion

**FIGURE 2:**
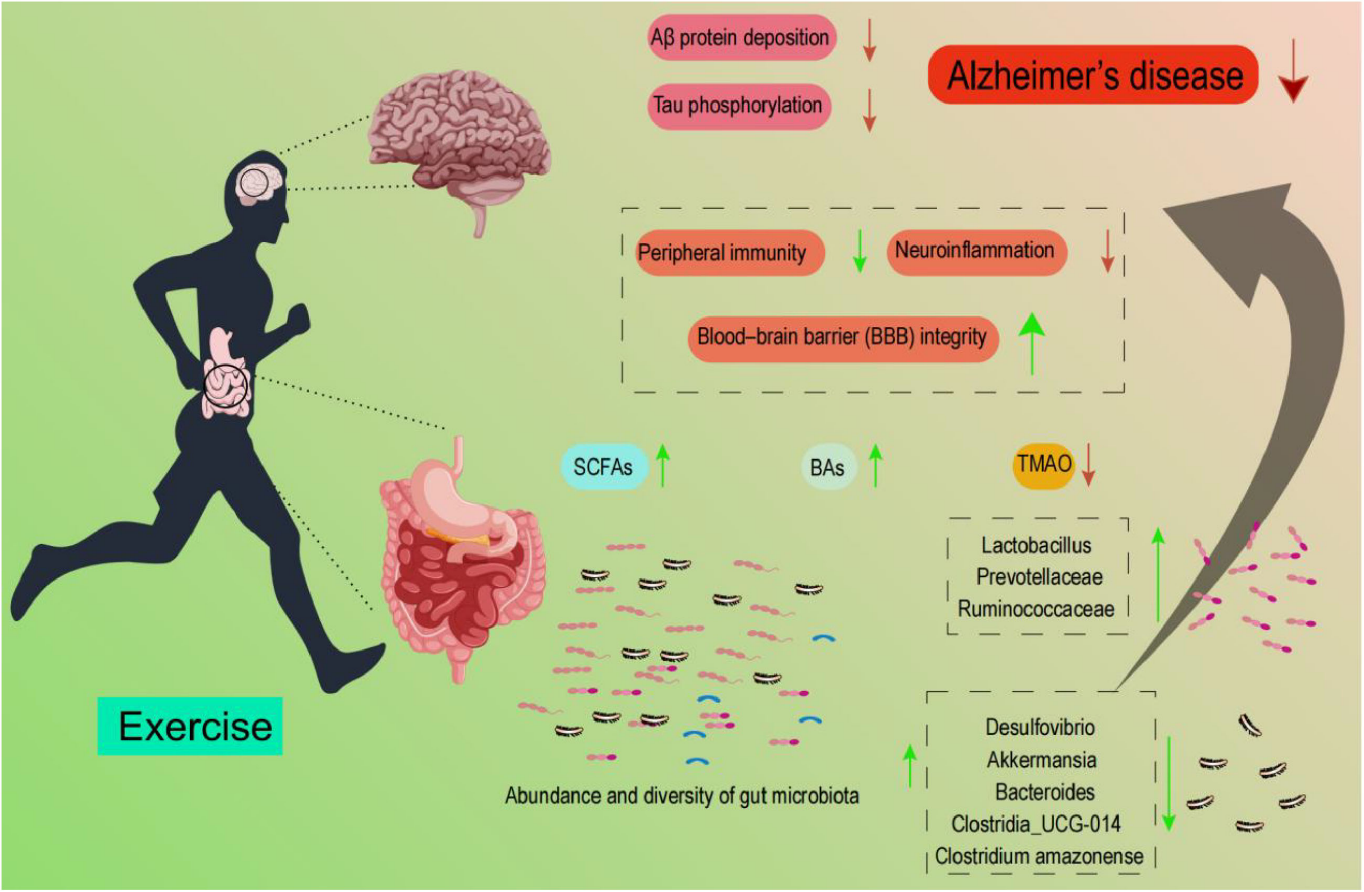
Schematic showing that exercise modulates gut-microbiota abundance/diversity and metabolites, thereby influencing peripheral immunity, neuroinflammation, and BBB integrity; these pathways are associated with reduced Aβ deposition and tau phosphorylation in AD. Listed taxa are illustrative, and their directions are context-dependent.

Numerous studies have highlighted the significant role of the gut microbiota in maintaining both physical and mental health, with dynamic interactions observed between physical exercise, the gut microbiota, and AD (Menezes and Shah, 2024; Fan et al., 2025). In this review, we explored the intricate relationship between the gut microbiota and AD, as well as the impact of exercise on gut microbiota composition. Furthermore, we provided a comprehensive analysis of the specific mechanisms by which exercise directly influences AD pathogenesis and how exercise may indirectly mitigate AD through modulation of the gut microbiota (Figure 2).

Beyond AD, the exercise–gut–brain axis has also been implicated in other neurodegenerative diseases. For instance, in Parkinson’s disease (PD), gut dysbiosis has been repeatedly documented and can promote α-synuclein aggregation and related pathophysiology; importantly, aerobic exercise in PD model mice has been shown to partially reverse microbiota disturbances and associate with behavioral and pathological improvements (Fan et al., 2022; Huang et al., 2023). In amyotrophic lateral sclerosis (ALS), recent clinical and mechanistic studies indicate that altered gut microbial profiles and their metabolites (including perturbations in SCFAs and other small molecules) are associated with disease progression and may affect motor neuron vulnerability (Fontdevila et al., 2024; Kaul et al., 2024). By contrast, the links in AD appear more systemic and indirect, mediated through circulating inflammatory factors, microbial metabolites, and blood–brain barrier integrity. This comparison underscores the unique systemic nature of AD pathology and reinforces the specific contribution of this review in focusing on Alzheimer’s disease. Building on this context, recent studies indicate that the gut microbiota not only contributes to pathological features of AD (e.g., Aβ deposition and tau hyperphosphorylation) but also represents a promising target for exercise-based interventions. Exercise can influence neuroendocrine regulation, modulate both peripheral and central immunity, and shape the production of microbial metabolites, thereby offering therapeutic potential to alleviate AD symptoms.

In real-world contexts, diet and exercise are inextricably linked lifestyle factors that jointly shape the gut–brain axis. Some intervention studies report that exercise increases the relative abundance of SCFA-producing taxa and elevates fecal butyrate (Varghese et al., 2024), with corresponding increases in butyrate observed in older, sedentary adults after a 24-week exercise intervention (Erlandson et al., 2021). Similarly, studies of athletes indicate a higher abundance of SCFA-producing taxa compared to sedentary controls (Fontana et al., 2023). Conversely, uncontrolled dietary variation may act as a confounder, obscuring the specific contribution of exercise (Kern et al., 2019). Future studies should therefore carefully account for diet–exercise interactions when designing interventions. Nonetheless, the present review deliberately focuses on exercise as a primary modifiable factor, with diet acknowledged as an important but separate domain requiring parallel investigation.

However, several aspects remain poorly understood, warranting further investigation. First, although the differential effects of exercise intensity and modality on gut health and various diseases have been confirmed, the specific influence of exercise on the gut–brain axis remains underexplored. Second, research focusing on exercise interventions targeting the gut microbiota to improve AD outcomes remains limited.

While existing evidence highlights two parallel streams—(i) exercise alters the gut microbiota and (ii) exercise ameliorates AD pathology (Aβ, tau)—the crucial causal link remains largely inferred rather than empirically established. To advance the field, future studies should adopt causal frameworks with clear criteria. For example, temporal precedence should be demonstrated so that exercise-induced microbiota/metabolite changes precede improvements in pathology (Gubert et al., 2020; Wang et al., 2022; Jin et al., 2023) dose–response relationships between exercise dose and graded microbiome/pathology changes should be sought (Kern et al., 2019; Varghese et al., 2024); mechanistic mediation needs validation via interventions that perturb the microbiota (e.g., fecal microbiota transplantation or antibiotic-mediated depletion) — several FMT studies show that transferring human or model-derived microbiota can alter amyloid/tau readouts in rodents (Kim et al., 2019; Grabrucker et al., 2023) and replicability across models and cohorts must be required (Gubert et al., 2020; Rojas-Valverde et al., 2023).

Third, current studies predominantly utilize male animal models, with limited attention to sex differences. Evidence from both animal and human studies indicates sex-dependent responses to exercise (Baker et al., 2010; Singh et al., 2014). Women may exhibit stronger HPA axis responses and greater BDNF sensitivity, and early-stage exercise interventions are associated with more robust neuroprotective effects than late-stage interventions. Therefore, future studies should adopt sex-balanced cohorts and stratified analyses by age, sex, and disease stage to better capture heterogeneity in exercise effects on the gut–brain axis.

**FIGURE 3:**
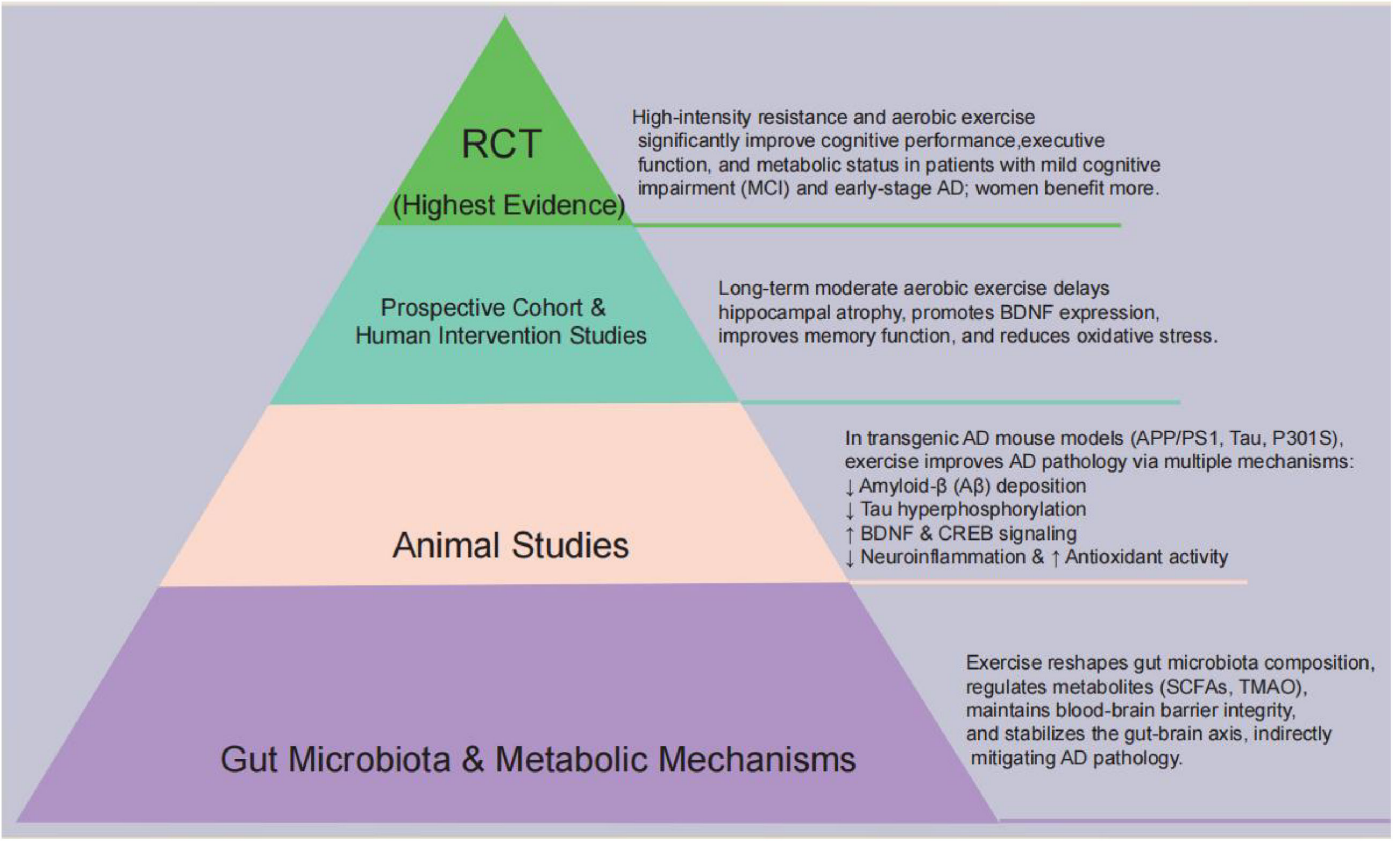
Evidence pyramid linking exercise, the gut–brain axis and AD: gut-microbiota and metabolic mechanisms at the base, followed by animal studies, prospective/human interventions, and RCTs; overall, aerobic and resistance exercise are associated with improved cognition and reduced AD pathology.

Moreover, conclusions are stratified according to the strength of evidence. findings from RCTs provide the strongest support that structured, moderate-intensity exercise improves cognitive outcomes and hippocampal volume in older adults. Prospective cohort studies offer additional support, although residual confounding cannot be excluded. Cross-sectional studies contribute correlational insights, while animal and *in vitro* studies remain hypothesis-generating. This evidence hierarchy underscores the translational potential of exercise while highlighting the need for large-scale, multi-level studies to confirm causality (Figure 3).

**FIGURE 4:**
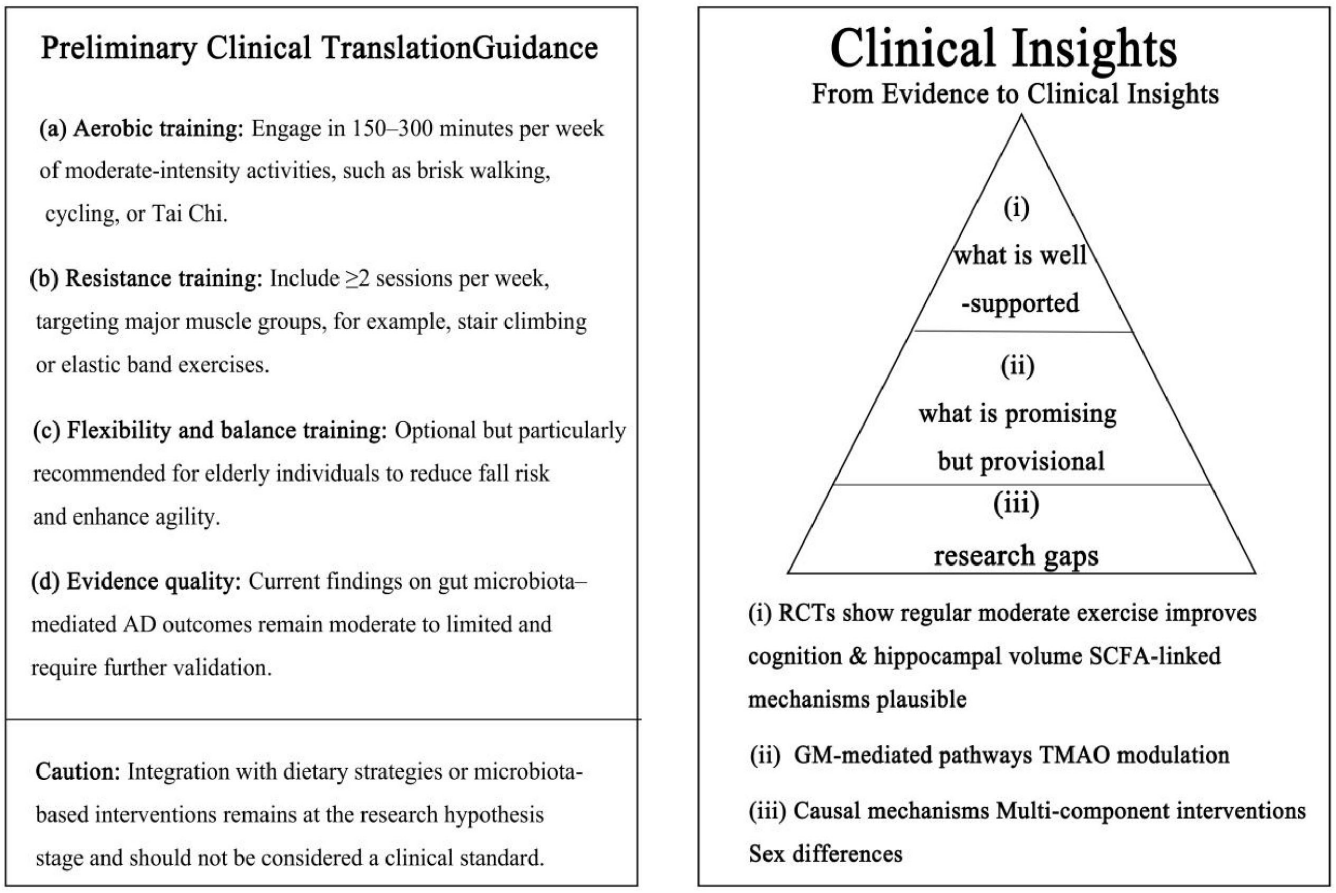
Clinical key insights and evidence hierarchy. Left: preliminary exercise guidance derived from reproducible human studies (aerobic, resistance, flexibility/balance; evidence quality and caution). Right: Evidence pyramid: (i) well supported—moderate-intensity exercise improves cognition and hippocampal volume; SCFA-linked mechanisms plausible; (ii) promising but provisional—gut microbiota-mediated pathways and TMAO modulation; (iii) research gaps—causal mechanisms, multi-component interventions, and sex differences. The figure aligns with the manuscript’s evidence tiers and avoids over-extrapolation.

Finally, integrating multi-component lifestyle interventions—including exercise, dietary modulation, and microbiota-targeted strategies—should be prioritized, alongside the application of multi-omics approaches and systems biology to elucidate mechanistic pathways. Large-scale, multi-center randomized controlled trials are essential to validate findings and improve translational relevance. Collectively, these refinements will enable the development of more personalized, evidence-based prevention and intervention strategies for AD (Figure 4).
